# Collaborative Solutions for Interference Management in GNSS-Based Aircraft Navigation

**DOI:** 10.3390/s20154085

**Published:** 2020-07-22

**Authors:** Mario Nicola, Gianluca Falco, Ruben Morales Ferre, Elena-Simona Lohan, Alberto de la Fuente, Emanuela Falletti

**Affiliations:** 1 Space and Navigation Technologies, LINKS Foundation, Via P. C. Boggio 61, 10138 Torino, Italy; gianluca.falco@linksfoundation.com (G.F.); emanuela.falletti@linksfoundation.com (E.F.); 2 Electrical Engineering unit, Tampere University, Korkeakoulunkatu 1, 33720 Tampere, Finland; ruben.moralesferre@tuni.fi (R.M.F.); elena-simona.lohan@tuni.fi (E.-S.L.); 3 GMV, Isaac Newton 11 P.T.M., 28760 Tres Cantos, Spain; afuente@gmv.com

**Keywords:** GNSS, jamming, spoofing, detection, classification, direction finding, source location, aircraft, civil aviation

## Abstract

Nowadays, the Global Navigation Satellite Systems (GNSS) technology is not the primary means of navigation for civil aviation and Air Traffic Control, but its role is increasing. Consequently, the vulnerabilities of GNSSs to Radio Frequency Interference, including the dangerous intentional sources of interference (i.e., jamming and spoofing), raise concerns and special attention also in the aviation field. This panorama urges for figuring out effective solutions able to cope with GNSS interference and preserve safety of operations. In the frame of a Single European Sky Air traffic management Research (SESAR) Exploratory Research initiative, a novel, effective, and affordable concept of GNSS interference management for civil aviation has been developed. This new interference management concept is able to raise early warnings to the on-board navigation system about the detection of interfering signals and their classification, and then to estimate the Direction of Arrival (DoA) of the source of interference allowing the adoption of appropriate countermeasures against the individuated source. This paper describes the interference management concept and presents the on-field tests which allowed for assessing the reached level of performance and confirmed the applicability of this approach to the aviation applications.

## 1. Introduction and State of the Art

Civil aviation and Air Traffic Control (ATC) are deeply tied to localization and navigation systems. Such systems are based on several technologies installed either on board or on the ground, including radio-beacons, RADAR, magnetic compasses, inertial navigation systems, and satellite positioning systems [1]. Global Navigation Satellite Systems (GNSSs) are complemented by their Wide-Area or Satellite-Based Augmentation Systems (WAAS, SBAS) to offer improved localization accuracy and an integrity framework to cope with flight-mode-dependent safety requirements [2]. Although the GNSS technology is not the primary means of navigation today for civil aviation and ATC, its role is increasing, starting from the General Aviation and Unmanned Aircraft; furthermore, several evolutions are expected in the next decade [1,3,4,5]. Indeed, new navigation and ATC concepts will be necessary in the perspective of a crowded sky in the near future, where millions of drones will share the airspace with manned aircraft; the Free Route Airspace (FRA) concept is an example of such new perspective [6]. In that panorama, continuous and accurate location of aircraft in the most crowded areas will enable safe and smart routing, collision avoidance, and fast emergency response; furthermore, it will allow location-based optimization of the communication links to offer broadband access for on-board entertainment [7].

The International Civil Aviation Organization (ICAO) and the European Organisation for Civil Aviation Equipment (EUROCAE) are working on shaping these trends, promoting the discussion about the evolution of the role of GNSS in aviation, while in parallel fostering the necessary technological advancement [3,4]. For example, ICAO released the concept of operations for the use of Dual-Frequency Multi-Constellation (DFMC) GNSS in aviation in April 2018 [8], while the Minimum Operational Performance Standard (MOPS) for GPS and Galileo on L1/E1 and L5/E5a frequency bands is under definition. DFMC GNSS is expected to replace the current single-frequency GPS L1-C/A in the future regulations for civil aviation. Other evolutionary concepts encompassing a prominent use of GNSS include Advanced Receiver Autonomous Integrity Monitoring (ARAIM) [9], Airbone Separation Assurance System (ASAS) [10], and multi-dimensional trajectory management [11].

On the other hand, the well-known vulnerabilities of GNSSs to Radio Frequency Interference (RFI) raise concerns and special attention also in the aviation field [1]. Facts witness an increasing number of reports of incidents of GPS outage on board of civil aircraft, especially in areas with political tensions (e.g., Southeast Mediterranean, Black Sea–Caspian Sea axes and Mideast-Canada and the USA via North Pole through Russian airspace) or nearby certain airports, according to the latest safety bulletin issued by Eurocontrol [12]. Once excluded events were caused by on-board GPS equipment failure, solar storms, military exercise, and the configuration of satellite constellations, and the Eurocontrol analysis concludes that the majority of the reported events could have been caused by intentional RFI, i.e., jamming. Nearby airports, also uninformed personal privacy devices could be the cause of GPS jamming. Consequently, jamming can be considered as a realistic and threatening kind of interference. On the other hand, spoofing is a more subtle and potentially even more dangerous threat, where an ensemble of counterfeit GNSS-like signals are injected in a victim receiver with the purpose of inducing a wrong positioning or timing provision of measure. Although spoofing attacks have not been reported yet in civil aircraft, their technical feasibility has been demonstrated and the potential danger in particular for unmanned aircraft is widely recognized [3].

This panorama, which has been alerted among others by International Air Transport Association (IATA) [13], urges for figuring out effective solutions able to cope with GNSS interference and preserve safety of operations: without the consolidation of such capability, the role of GNSS in safety operations might be controversial. For this reason, plans to mitigate the effects of RFI are under development [14], and initiatives have been started in Europe to foster the research and development on these topics—for example the Single European Sky Air traffic management Research (SESAR) Evolutionary Research (ER) program and the Horizon 2020 Research and Innovation program [15]. Thus far, initiatives have been focused on: detection of interference on-board helicopters using the existing GNSS antenna, which provides jamming detection without localization [16], localization of jamming interference using flight tracking from Automatic Dependent Surveillance-Broadcast (ADS-B), which is under evaluation [17], localization of jamming interference using on-board Controlled Radiation Pattern Antenna (CRPA) antennas, which requires new complex antennas on-board [18], and some others. None of them suggests using existing omnidirectional GNSS antennas to detect and localize jamming and spoofing.

The objective of this paper is to present the results of an on-field test campaign of a novel, effective, and affordable concept of GNSS interference management for civil aviation, developed under a SESAR ER initiative [19]. This new interference management concept relies on known techniques of detection and localization of jamming and spoofing, which have been adapted to the restrictions imposed by the target environment, i.e., using a minimum number of omnidirectional antennas on the fuselage, with the minimum impact on the current on-board equipment. This concept is founded on a set of capabilities: (i) to “seamlessly” cope with different categories of interference, namely various types of jamming signals and spoofing signals; (ii) to raise early warnings to the on-board navigation system about the detection of interfering signals and their classification (e.g., jamming or counterfeit signals); (iii) to estimate the Direction of Arrival (DoA) of the source of interference, thanks to the use of three antennas placed on the aircraft body; (iv) to enable collaborative solutions for the localization of the source of interference, exploiting multiple DoA measurements along the time and from different aircraft; (v) to leverage as much as possible on existing or realistically expected aircraft equipment, with the target of minimizing the aircraft retrofit and making technology acceptance easier.

**Figure 1:**
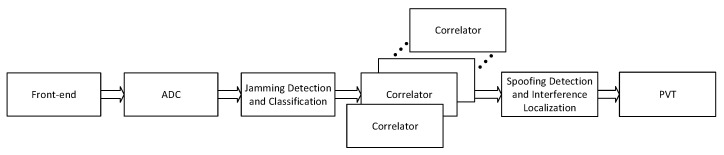
Block diagram of the proposed solution. The pre-correlation block is indicated as ‘Jamming detection and classification’; the post-correlation block is the ‘Spoofing detection and interference localization’.

**Figure 2:**
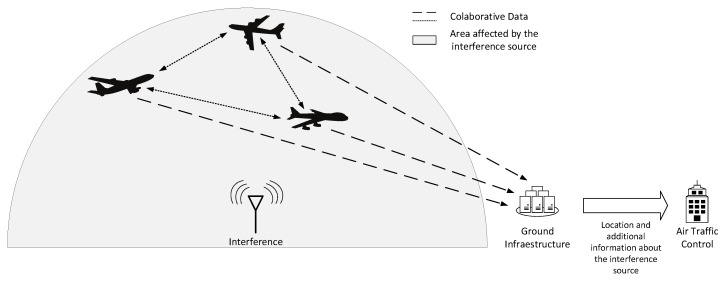
Scenario diagram of a collaborative interference management solution with joint on-board and on-the-ground processing.

The novelty of the proposed concept is two-fold, both on board and on the ground. On board, the signal processing architecture is developed as an external two-blocks add-on of existing GNSS receivers, as sketched in Figure 1: in the *pre-correlation block*, the received radio frequency signal from each antenna is pre-processed before entering the receiver operations, in order to detect and classify a possible jamming signal and to estimate its DoA. In the *post-correlation block*, the receivers’ outputs in the form of code and carrier pseudorange measurements are used to detect possible counterfeit (spoofed) signals in the ensemble processed by the receivers and to determine their DoA. On the ground, a hybridization server implements the collaborative interference management: it receives measurements from all the aircraft in the area regarding the presence of interference (e.g., interference detection flags, identified interference classes, raw carrier, and code measurements, etc.) and combines the cooperative information through a hybridization mechanism, e.g., based on machine learning or particle filtering approaches. A schematic block diagram of such a collaborative approach is depicted in Figure 2.

With respect to [19], where this interference management concept was introduced for the first time with the support of preliminary in-lab simulation results, this paper completes and formalizes the description of the detection and direction finding methods for both the jamming and the spoofing interferences. Then, the proposed methods have been tested with an on-field test campaign: the analysis of the obtained results allows for validating the new interference management concept for the civil aviation while assessing the obtained level of performance.

The on-field tests, together with the practical implementation of the interference detection and direction finding algorithms which takes into account the existing or realistically expected aircraft equipment, are the main contributions of this paper with respect to existing sources in literature. Indeed, the context of active GNSS interference management from on-board aircraft reusing omnidirectional navigation antennas is new by itself in the civil aviation field. To the best of the authors’ knowledge, no literature exists that addresses in an integrated way the various interference types encountered in GNSS and that explicitly deals with the stages to counteract this interference (for example, [1] presents a comprehensive review of the literature about intentional interferences). It must be added that, in such conditions, a fair comparison with algorithms existing in literature is not possible due to the novelty of the strategy, i.e., interference detection and localization with systems on-board the aircraft instead of existing systems deployed on-ground. In addition, the novelty is focused more in the adoption in the aviation scenario and the adaptation to existing infrastructures than in large scale novelties at an algorithmic level.

The paper is organized in Seven sections: Section 2 introduces the concept and possible architectures of the novel collaborative interference management approach; Section 3 discusses the signal processing algorithms proposed for the jamming detection and classification; Section 4 is devoted to the algorithms to detect spoofing and identify the direction of arrival of the counterfeit signals; Section 5 describes the campaign of trials in open field, with the results analyzed and commented on in Section 6; Section 7 summarizes the conclusions and draws the perspective of evolution for the concepts presented in the paper. Finally, Appendix A lists all the acronyms used through the text of the paper.

## 2. Novel Concepts for Interference Management: From Autonomous to Collaborative Solutions

The integrated GNSS interference management aims to provide an accurate position of the interference source sensed on-board the aircraft and to report the information to ATC. Depending on the complexity of the system, two modes of operation have been defined to provide accurate localization:

- Detection and Autonomous Localization (D&AL)
- Detection and Collaborative Localization (D&CL)

### 2.1. Detection and Autonomous Localization

The concept of autonomy here is based on the idea that the aircraft relies only on the data recorded on-board to localize the interference source. During nominal operation (detection), the aircraft is continuously monitoring the presence of jamming or spoofing interference, using specific algorithms to detect each type of attack. When an interference is detected, the aircraft automatically starts the localization. At every epoch, the aircraft estimates the DoA of the interfering signal, using the corresponding algorithm for each type of interference. In addition, the aircraft integrates the localization computed at each epoch along the trajectory where the interference is affecting. It provides an accurate localization of the interference thanks to benefit from the movement of the aircraft with respect to the interference source. As soon as the aircraft has estimated a ‘reliable’ position of the interference source, the ATC has to be reported with an alert and some additional information. The information reported by the aircraft includes the type of interference, the estimated position of the interference source, a time-tag, the error in the estimated position and the estimated affected volume (radius of the affected area at certain flight level). For the sake of automation and with regard to the low data required to transmit the information from the aircraft to the ATC, it is recommended to use a data link (e.g., ADS-B Mode S 1090 MHz Extended Squitter).

### 2.2. Detection and Collaborative Localization

This mode is collaborative in the sense that the localization estimated by multiple affected aircraft is integrated on-ground, potentially achieving a more accurate localization of the source. During nominal operation (detection), each aircraft is continuously monitoring the presence of interference, as in the previous mode D&AL. The main difference of D&CL mode with respect to mode D&AL is the need of a ground infrastructure. The estimated DoAs of the interfering signal and associated estimation error statistics computed by each aircraft at every epoch are transmitted to the ground infrastructure, which can compute a better localization of the source thanks to the multiple sources of information (i.e., multiple aircraft affected by the interference).

It is interesting to notice that, in D&CL mode, aircraft transmit the information to the ground infrastructure, whereas in D&AL mode it is transmitted to ATC. Nevertheless, the same information is transmitted in both modes and therefore the same data-link can be used.

### 2.3. Airborne Implementation

**Figure 3:**
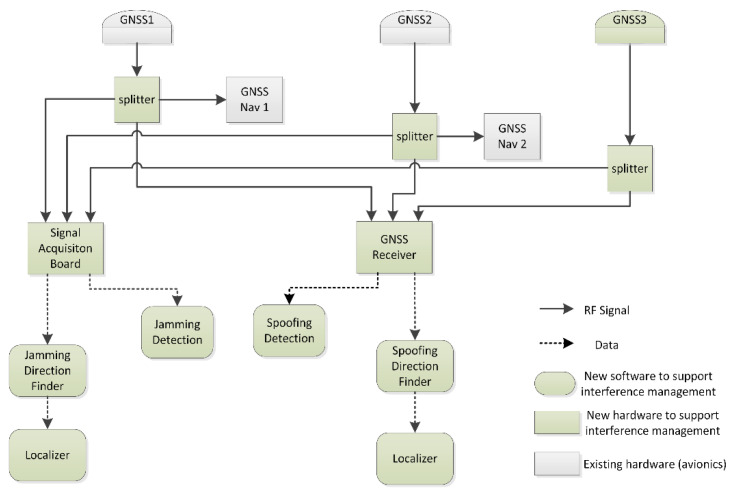
High level airborne system architecture.

One of the most important constraints assumed in this work is to minimize the installation of additional equipment on-board the aircraft. Taking this into account, the elements that require modification are:

*GNSS antennas layout:* Currently, two GNSS omnidirectional antennas are normally available for navigation and placed on top of the fuselage. Moreover, an additional third omnidirectional antenna is required for interference localization. Layout of a right triangle with baselines between 1 and 3 m is a suitable configuration for the GNSS antennas to support detection and localization of both jamming and spoofing.
*Data processing:* Additional hardware and software is needed on-board to process the signal from the GNSS antennas and to implement the detection and localization algorithms. Figure 3 shows the block scheme of the airborne system architecture, highlighting the additional hardware required.

### 2.4. Interference Localization: Model of the Problem

The main benefit of the interference source localization is the capability to estimate the position of the interference source, based on the measured angles obtained through the DoA finding techniques defined for jamming and spoofing. In this section, the D&AL approach is considered, but the analysis can be straightforwardly extended to D&CL. The variables defining the model of the problem are:

- $u_{x},\;u_{y}$: Unitary vectors along the *x* and *y* reference axes;
- $p=p_{x}u_{x}+p_{y}u_{y}$: Interference source location vector (unknown, to be estimated), it is assumed to be fixed during the collection of measurements;
- $\hat{p}$: Estimate of the interference source location vector;
- $K_{\hat{p}}=\mathrm{Cov}\left(\hat{p}\right)$: Covariance matrix of the estimated location, expressing the statistics of the location estimation error;
- $r\left[n\right]=r_{x}\left[n\right]u_{x}+r_{y}\left[n\right]u_{y}$: Observer (i.e., aircraft) position, at each time epoch *n* (known from the normal aircraft operations);
- θ[n]: Azimuth angle (DoA) between the observer and the interference source (unknown, estimated by signal processing algorithms);
- w[n]: Random variable that models the measurement error on θ[n] (unknown).
- $\tilde{\theta }\left[n\right]=\theta \left[n\right]+w\left[n\right]$: Measure of the azimuth angle, affected by a measurement error (measured).

It is assumed that *N* measurements are available and then two column vectors are added to the defined symbols

$\theta ={\left[\begin{array}{cccc} \theta [0] & \theta [1] & \cdots & \theta [N-1] \end{array}\right]}^{T}$: Vector made of *N* unknown azimuth angles;
$\tilde{\theta }={\left[\begin{array}{cccc} \tilde{\theta }\left[0\right] & \tilde{\theta }\left[1\right] & \cdots & \tilde{\theta }\left[N-1\right] \end{array}\right]}^{T}$: Vector made of *N* estimated azimuth angles.

Such measurements are generated either *(i)* from the same source (aircraft) in different time instants (D&AL), or *(ii)* from different sources (aircraft) in approximately the same time instant (D&CL).

The problem of estimating $\hat{p}$ and $K_{\hat{p}}$ from the set of measurements $\tilde{\theta }\left[n\right]$ has been modeled as follows:

(1) $$\begin{array}{cc} \theta [n] & =\mathrm{atan}\left(\frac{p_{y}-r_{y}\left[n\right]}{p_{x}-r_{x}\left[n\right]}\right) \end{array}$$

(2) $$\begin{array}{cc} \nabla \theta [n] & =\frac{\partial }{\partial p_{x}}\theta \left[n\right]u_{x}+\frac{\partial }{\partial p_{y}}\theta \left[n\right]u_{y}=\frac{1}{∥p-r[n]∥}{\left[-\sin\left(\theta [n]\right)\;\cos\left(\theta [n]\right)\right]}^{T} \end{array}$$

Considering *N* angular measurements available, then the above equation becomes

(3) $$\nabla \Theta_{*,n}=\frac{1}{∥p-r[n]∥}\left[\begin{array}{c} -\sin\left(\theta [n]\right)\\ \cos\left(\theta [n]\right) \end{array}\right]$$

where $\nabla \Theta_{*,n}$ is the *n*-th column of the 2×N matrix ∇Θ.

The problem has also been solved using Maximum Likelihood Estimation (MLE) assuming that w[n] is a white noise truncated Gaussian random variable between ±π, obtaining more accurate estimations of $\hat{p}$. MLE estimates $\hat{p}$ maximizing the likelihood function $f\left(\tilde{\theta }|p\right)$:

(4) $$\hat{p}=\underset{p}{\mathrm{argmax}}f\left(\tilde{\theta }|p\right)$$

(5) $$f\left(\tilde{\theta }|p\right)=\frac{1}{\sqrt{{\left(2\pi \right)}^{N}\left|K_{\hat{p}}\right|}}\exp\left[-\frac{1}{2}{\left(\tilde{\theta }-\theta \right)}^{T}K_{\hat{p}}\left(\tilde{\theta }-\theta \right)\right]$$

where $K_{\hat{p}}$ can be expressed as a N×N diagonal matrix

(6) $$K_{\hat{p}}=\mathrm{diag}\left[\begin{array}{cccc} \frac{1}{\sigma_{w[0]}^{2}} & \frac{1}{\sigma_{w[1]}^{2}} & \cdots & \frac{1}{\sigma_{w[N-1]}^{2}} \end{array}\right]$$

Working with the log-likelihood is more convenient:

(7) $$\hat{p}=\underset{p}{\mathrm{argmax}}\left(\ln f\left(\tilde{\theta }|p\right)\right)=\underset{p}{\mathrm{argmin}}\left(2J_{ML}\left(\theta \right)\right)=\underset{p}{\mathrm{argmin}}\left(J_{ML}\left(\theta \right)\right)$$

where

(8) $$\ln f\left(\tilde{\theta }|p\right)=-\frac{1}{2}\ln\left({\left(2\pi \right)}^{N}\left|K_{\hat{p}}\right|\right)-\frac{1}{2}{\left(\tilde{\theta }-\theta \right)}^{T}K_{\hat{p}}\left(\tilde{\theta }-\theta \right)$$

(9) $$J_{ML}\left(\theta \right)={\left(\tilde{\theta }-\theta \right)}^{T}K_{\hat{p}}\left(\tilde{\theta }-\theta \right)=\sum_{n=0}^{N-1}\frac{1}{2\sigma_{w[n]}^{2}}{\left(\tilde{\theta }\left[n\right]-\theta \left[n\right]\right)}^{2}$$

In order to find the minimum of $J_{ML}\left(\theta \right)$, the gradient has to be equal to 0. Gradient of cost function is shown in Equation (10), where ∇Θ is defined in Equation (3):

(10) $$\nabla J_{ML}\left(\theta \right)=-2\nabla \Theta K_{\hat{p}}\left(\tilde{\theta }-\theta \right)=0$$

However, Equation (10) does not have analytical solution. Therefore, Equation (7) must be solved using numerical procedures with iterative algorithms based on the following formula, where $d^{\left(i\right)}$ characterizes the direction of change in the parameter space and $s^{\left(i\right)}$ controls the amount of change:

(11) $$p^{\left(i+1\right)}=p^{\left(i\right)}+s^{\left(i\right)}d^{\left(i\right)}=p^{\left(i\right)}-s^{\left(i\right)}\nabla J_{ML}\left(\theta \right)$$

As we are looking for the minimum of $J_{ML}\left(\theta \right)$, then $d^{\left(i\right)}=-\nabla J_{ML}\left(\theta \right)$.

Several numerical solutions have been compared (e.g., steepest descent, Newton–Raphson, Gauss–Newton), but the best results are obtained by solving with Levenberg–Marquardt method [20]. This method can be thought of as a combination of steepest descent and the Gauss–Newton method. When the current solution is far from the correct one, the algorithm behaves like a steepest descent method, slow but guaranteed to converge. When the current solution is close to the correct solution, it becomes a Gauss–Newton method. Levenberg–Marquardt is based on this formula, where *i* and i+1 are the indexes of two consecutive steps of the algorithm.

(12) $$p^{\left(i+1\right)}=p^{\left(i\right)}+{\left[\left(\nabla \Theta^{\left(i\right)}\right)K_{\hat{p}}{\left(\nabla \Theta^{\left(i\right)}\right)}^{T}+\lambda \left(\left(\nabla \Theta^{\left(i\right)}\right)K_{\hat{p}}{\left(\nabla \Theta^{\left(i\right)}\right)}^{T}\right)\right]}^{-1}\left(\nabla \Theta^{\left(i\right)}\right)K_{\hat{p}}\left(\tilde{\theta }-\theta \right)$$

The parameter λ is initialized to a fixed value and then updated in each iteration as described in the pseudo-code implementation in [20].

## 3. Methods for Jamming Detection and Classification

The techniques for interference detection, localization, and classification can be applied at different stages of the GNSS receiver chain: Front-end (e.g., Automatic Gain Control (AGC) [21]), pre-correlation (e.g., power detectors such as Time Power Detector (TPD)/Frequency Power Detector (FPD) [22,23]), post-correlation (e.g., Carrier-to-Noise Ratio ($C/N_{0}$) monitoring [24]) or at navigation level (e.g., Sum of Squares detector [25,26]). This section focuses on techniques applied before correlation. With these techniques, one can determine the presence of interference earlier than with the rest of techniques; the only needed input is the raw signal received by the GNSS antenna; no other prior information is needed, such as number or Space Vehicle (SV) number of satellites in view. The chosen detection techniques as well as a description of the classification method are detailed next.

The first technique is the so-called AGC detector [21]. This technique monitors the AGC, which is located at the front-end. AGC is in charge of maintaining the control of the power of the incoming signal to provide an appropriate power for the signal quantizer, in order to minimize the quantization losses. The AGC of a GNSS receiver operates at the ambient noise levels, since the received signal power is extremely low. In the presence of interference, the AGC decreases its gain to keep the AGC output signal level stable and avoid large fluctuations. By monitoring this gain and establishing a threshold, the GNSS receiver can determine if an interference is present, as it is described in the following rule:

(13) $${AGC}_{\mathrm{level}}=\left\{\begin{array}{cc} >\gamma & \mathrm{Interference}\;\mathrm{detected}\\ <\gamma & \mathrm{Interference}\;\mathrm{free} \end{array}\right.$$

where the test statistic is represented by ${AGC}_{\mathrm{level}}$, which is compared with a certain threshold γ. If ${AGC}_{\mathrm{level}}$ is larger than γ, it is determined that an interference is present. Otherwise, no interference scenario is established.

The following described techniques are used at the pre-correlation stage too. FPD and TPD (the latter also called Power Law Detector (PLD) or energy detector) measure the received signal energy over a short period of time; the measured power is then compared with a suitable threshold. The test statistics, in time and frequency domain, are defined in Equations (14) and (15), respectively:

(14) $${TPD}_{\mathrm{level}}=\frac{1}{JN}\sum_{j=1}^{J}\sum_{n=1}^{N}{\left|r[n+(j-1)N]\right|}^{2\nu },$$

(15) $${FPD}_{\mathrm{level}}=\frac{1}{JK}\sum_{j=1}^{J}\sum_{k=1}^{K}{\left|R[k+(j-1)K]\right|}^{2\nu },$$

where |r[n]| is the absolute value of the raw GNSS signal received by the antennas, N is the number of samples of the considered short interval, J is the number of short intervals under the observations (thus the signal is observed in total over JN samples), and ν is a positive number that determines the power-law, e.g., ν=1 for the square-law detector and ν=0.5 for the amplitude detector. R(k) is the Fourier transform of the |r(n)| signal, and K is the number of frequency samples over which we compute the signal power (JK is the overall considered frequency window).

The third detector type is based on the information given by the entropy of the received signal, which is defined as the measure of the average information content per source symbol. The entropy can be calculated as

(16) $${Entropy}_{\mathrm{level}}=-\sum_{j=1}^{N}\;p_{j}\;\log_{b}\;p_{j},\;$$

where $p_{j}$ is the probability of the occurrence of character number j from a given stream of characters and b is the base of the algorithm used. Equation (16) shows how to determine if only GNSS signal is received (which can be considered as white noise) or if GNSS plus jamming signals are received together. The entropy is at a maximum when the jammer is not present, since the probability of the different source symbols is minimum (they are random). In case of a clear contribution of a specific signal (interference), the entropy drops considerably compared with the maximum achievable entropy, due to the fact that a coherent signal is found.

The fourth considered detector is based on the Kurtosis measurement. The Kurtosis measures how much the tails of a distribution differ from the tails of a normal distribution—or, in other words, it identifies whether the tails of a given distribution contain extreme values (as, for example, the tails of a Gaussian distribution). Kurtosis is defined as

(17) $${Kurtosis}_{\mathrm{level}}=\frac{\frac{1}{N}\sum_{n=1}^{N}{\left(r\left(n\right)-\mu_{r}\right)}^{4}}{{\left(\frac{1}{N}\sum_{n=1}^{N}{\left(r\left(n-\right)\mu_{r}\right)}^{2}\right)}^{2}},$$

where $\mu_{r}=\frac{1}{N}\sum_{n=1}^{N}r\left(n\right)$ is the mean of the signal r(n). In the absence of jamming, the Kurtosis is close to 3 (Gaussian distribution). In the presence of a jamming signal, the Kurtosis may deviate from value 3 with a deviation which depends on the type of jamming.

Finally, the last detector described in this paper is based on the Teager–Kaiser (TK) operator [27]. TK measures the energy of a certain signal. The discrete TK operator of a complex valued signal is given by [28]

(18) $${TK}_{\mathrm{level}}=\sum_{n=1}^{N}r^{*}\left[n\right]r\left[n\right]-\frac{1}{2}\left[r^{*}\left[n+1\right]r\left[n+1\right]-r\left[n-1\right]r^{*}\left[n-1\right]\right],$$

Besides jamming detection, classification of jamming signals is another important aspect. Not so many efforts have been put in the existing literature so far on the classification compared with detection. In this paper, a solution for jamming classification is also addressed, by using the different features the interference signal introduces in the received GNSS signal. Jamming classification can be split in the following steps [29]:

1. Signal Time-Frequency (TF) Transform: A certain TF transform is applied to the raw signal received by the GNSS antenna. Here, the chosen TF transform is the spectrogram transform, due to its relative low complexity and high accuracy.
2. Image generation: After the TF transform, an image is generated and stored. A library with a huge number of images is created as a training database. The images are labeled and divided as training and testing data sets for further use.
3. Features extraction: Before applying any classification algorithm, an image feature extraction procedure is applied. This is done in order to obtain features from the set of images that can be used to train the algorithm.
4. Algorithm training: The extracted features are used in order to train the classifier. The chosen algorithm was Support Vector Machine (SVM) due to its easy parameter setting and high performance for image classification. The training procedure is called ‘supervised training’, since the images used for training are previously labeled. With this procedure, the algorithm learns which features are related to each interference type.
5. Algorithm evaluation: finally, after the algorithm is trained, it is ready for using testing images (which are not labeled) in order to check the accuracy of the classifier.

The mentioned methods have been applied both in the lab on synthetic signals, as presented in [19], and on live signals recorded during the open-field test campaign described hereafter. The results of the latter test campaign are reported in Section 6.1 of this paper.

## 4. Methods for Spoofing Detection and Direction Finding

Many kinds of spoofing attack exist: they differ in the level of complexity/cost of realization at the attacker side, and pose different levels of threat to the target receiver [30]. Amongst them, the most realistic spoofing attacks are those based on a single transmitting antenna, whereas the use of multiple transmitting antennas, typical of the so-called *advanced* or *sophisticated* spoofing, is regarded as a high cost, high complexity, and less common type of attack [30]. The realistic assumption of a single transmitting antenna at the attacker side and the availability of multiple antennas at the receiver side make possible a spoofing detection based on the estimation of the DoA of the received signal [25,31,32]: if the DoA is not compatible with the expected satellite positions, then the existence of a counterfeit transmission is detected. The DoA evaluation is based on the post-correlation observables produced by the receivers.

Indeed, the code and carrier phase pseudoranges produced by the receivers for each Pseudo Random Noise (PRN) code in view [33], differenced over each antenna pair i,j is expressed by Equations (19) and (20) as

(19) $$\Delta \rho_{ij}^{\left(m\right)}=\Delta d_{ij}^{\left(m\right)}+c\Delta T_{ij}+\Delta \epsilon_{\rho ,ij}^{\left(m\right)}$$

(20) $$\Delta \phi_{ij}^{\left(m\right)}=\Delta d_{ij}^{\left(m\right)}+c\Delta T_{ij}+\lambda_{f}\Delta N_{ij}^{\left(m\right)}+\Delta \epsilon_{\phi ,ij}^{\left(m\right)}$$

where $\Delta \rho_{ij}^{\left(m\right)}$ and $\Delta \phi_{ij}^{\left(m\right)}$ denote the Single Difference (SD) code and carrier phase pseudoranges in meters for the m-th source, $\Delta d_{ij}^{\left(m\right)}$ is the SD ij geometric range (i.e., SD distance of the m-th source from the i,j-th antennas), c is the speed of the light, $\Delta T_{ij}$ is the SD ij clock error, $\lambda_{f}$ is the wavelength, $\Delta N_{ij}^{\left(m\right)}$ is the SD ij carrier phase integer ambiguity, $\Delta \epsilon_{\rho ,ij}^{\left(m\right)}$ and $\Delta \epsilon_{\phi ,ij}^{\left(m\right)}$ are differential noise terms accounting for residual not modeled errors, including thermal noise and multipath [33]. In the following, the measurements are assumed to be synchronized, then $\Delta T_{ij}\approx 0$.

The geometric range difference between the satellite and the antennas $\Delta d_{ij}^{\left(m\right)}$ contains a geometrical term, which depends on the DoA of the m-th source with respect to the antennas position (angle $\theta^{\left(m\right)}$): it is the component, along the ij baseline, of the orthogonal projection of the unitary vector $x^{\left(m\right)}$ representing the signal DoA:

(21) $$\Delta d_{ij}^{\left(m\right)}=g_{ij}^{T}x^{\left(m\right)}=D\cos\left(\theta^{\left(m\right)}\right)$$

where $g_{ij}$ is the geometrical vector describing the relative position of the antenna j with respect to the antenna i (baseline ij) and D=$\left|g_{ij}\right|$. In Equation (21), the DoA of the signal is represented both as an angle (θ) and as a unitary vector (x). This geometrical term can be the basis of a possible spoofing countermeasure because:

- if more signals share the same geometrical term, they are likely produced by the same source, so they are not genuine (detection);
- the common DoA of such counterfeit signals can be extracted from the common geometrical term (direction finding).

Taking this into account, it is possible to figure out a procedure which combines the detection of the spoofing and the DoA evaluation (Spoofing Detection and Direction Finding (SpDDF)):

1. the carrier phase observables produced at each epoch by three receivers, connected to three antennas properly spaced each other, enter the detection module;
2. the detection algorithm forms the SDs and Double Differences (DDs) for each antenna and signal pair at each measurement epoch; it monitors its detection metric computed from the DD measurements;
3. if a set of signals is declared ‘spoofed’, then the Direction Finding algorithm is activated on the SD code and carrier phase measurements for the current epoch and the DoA is estimated;
4. the SpDDF procedure continues to the next epoch.

**Figure 4:**
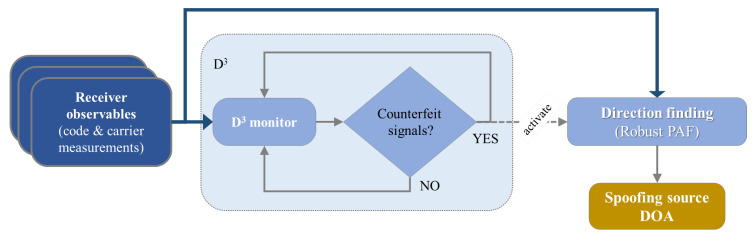
Principle of the Spoofing Detection and Direction Finding (SpDDF) procedure.

Figure 4 reports a block scheme representing the steps of the SpDDF procedure.

### 4.1. Spoofing Detection

The algorithm used for the spoofing detection, named Dispersion of Double Differences ($D^{3}$), derives from the Sum of Squares [25] improved as presented in [26]. A detailed performance analysis of the algorithm is available in [34]. The $D^{3}$ algorithm is based on the DDs of pairs of carrier phase measurements, along the ij baseline:

(22) $$\nabla \Delta \varphi_{ij}^{\left(m\right)}=\frac{1}{\lambda_{f}}\left(\Delta \phi_{ij}^{\left(m\right)}-\Delta \phi_{ij}^{\left(0\right)}\right)=\frac{\Delta d_{ij}^{\left(m\right)}-\Delta d_{ij}^{\left(0\right)}}{\lambda_{f}}+\Delta N_{ij}^{\left(m\right)}-\Delta N_{ij}^{\left(0\right)}+\Delta \epsilon_{\varphi ,ij}^{\left(m\right)}-\Delta \epsilon_{\varphi ,ij}^{\left(0\right)}$$

expressed in number of cycles, where the superscript (0) indicates the signal taken as a reference. In order to remove the effect of the DD integer ambiguity, the fractional part of Equation (22) is considered [25], i.e.,:

(23) $$\mathrm{frac}\left(\nabla \Delta \varphi_{ij}^{\left(m\right)}\right)=\mathrm{frac}\left(\frac{\Delta d_{ij}^{\left(m\right)}-\Delta d_{ij}^{\left(0\right)}}{\lambda_{f}}+\Delta \epsilon_{\varphi ,ij}^{\left(m\right)}-\Delta \epsilon_{\varphi ,ij}^{\left(0\right)}\right)=\mathrm{frac}\left(\nabla \Delta d_{ij}^{\left(m\right)}+\nabla \Delta \epsilon_{\varphi ,ij}^{\left(m\right)}\right)$$

where $\nabla \Delta d_{ij}^{\left(m\right)}$ and $\nabla \Delta \epsilon_{\varphi ,ij}^{\left(m\right)}$ indicate the DD of the geometric term and the error term, respectively. In Equation (22), $\Delta N_{ij}^{\left(m\right)}-\Delta N_{ij}^{\left(0\right)}$ is made of an integer number of carrier cycles, then it has no impact on the evaluated fractional part and has been deleted from Equation (23). Moreover, depending on the baseline geometry, the term $\nabla \Delta d_{ij}^{\left(m\right)}$ is made of an integer number of carrier cycles $\nabla \Delta d_{ij,int}^{\left(m\right)}$ and a fractional part $\nabla \Delta d_{ij,frac}^{\left(m\right)}$. Again, the term $\nabla \Delta d_{ij,int}^{\left(m\right)}$ is removed by the frac operator, and then only the term $\nabla \Delta d_{ij,frac}^{\left(m\right)}$ is used for the spoofing detection. If the noise term $\nabla \Delta \epsilon_{\varphi ,ij}^{\left(m\right)}$ is small with respect to $\nabla \Delta d_{ij,frac}^{\left(m\right)}$, Equation (24) follows:

(24) $$\mathrm{frac}\left(\nabla \Delta \varphi_{ij}^{\left(m\right)}\right)=\mathrm{frac}\left(\nabla \Delta d_{ij,frac}^{\left(m\right)}+\nabla \Delta \epsilon_{\varphi ,ij}^{\left(m\right)}\right)\approx \nabla \Delta d_{ij,frac}^{\left(m\right)}$$

When the receiver locks to counterfeit signals, the related fractional DDs cluster around a common value, whereas the values obtained for the authentic signals differ depending on the actual azimuth of the satellite. This behavior is the basis of $D^{3}$ for discriminating between authentic and counterfeit signals. Details about how the $D^{3}$ algorithm grants the robustness towards noisy measurements and copes with the possible coexistence of measurements from both the counterfeit and the authentic signals can be found in [26,34]. It must be noticed that one baseline, i.e., one antenna pair, is enough to execute the $D^{3}$ algorithm, but the presence of additional antennas can be exploited to reach more reliable results.

### 4.2. Direction Finding

Once a subset of M counterfeit signals is identified, then an adaptation and redundant implementation of the Precise and Fast (PAF) algorithm shown in [35] is employed to estimate the azimuth of the spoofing source with respect to the antenna frame [36]. The formulation adopted here employs the SD code and carrier phase equations of all the same-source signals along two baselines (ij)=(12) and (ij)=(13). The equations for the m-th counterfeit signal are:

(25) $$\begin{array}{ccc} \left[\begin{array}{c} \Delta \rho_{12}^{\left(m\right)}\\ \Delta \rho_{13}^{\left(m\right)} \end{array}\right] & = & \left[\begin{array}{c} g_{12}^{T}\\ g_{13}^{T} \end{array}\right]x+\left[\begin{array}{c} \Delta \epsilon_{\rho ,12}^{\left(m\right)}\\ \Delta \epsilon_{\rho ,13}^{\left(m\right)} \end{array}\right]\\ \left[\begin{array}{c} \Delta \phi_{12}^{\left(m\right)}\\ \Delta \phi_{13}^{\left(m\right)} \end{array}\right] & = & \left[\begin{array}{c} g_{12}^{T}\\ g_{13}^{T} \end{array}\right]x+\lambda_{f}\left[\begin{array}{c} \Delta N_{12}^{\left(m\right)}\\ \Delta N_{13}^{\left(m\right)} \end{array}\right]+\left[\begin{array}{c} \Delta \epsilon_{\phi ,12}^{\left(m\right)}\\ \Delta \epsilon_{\phi ,13}^{\left(m\right)} \end{array}\right] \end{array}$$

where the vector x is the common DoA of all the counterfeit signals. The above set of equations taken for the M counterfeit signals (*multi-satellite problem*) consists of a system of 4M equations and (2+2M) unknowns, i.e., the bi-dimensional vector x and the 2M SD integer ambiguities. The system has rank equal to the number of unknowns, then it is overdetermined but consistent ∀M>1 and a Least Squares float solution exists. Once obtained the float solution, the vector of ambiguities can be constrained to integer values by using an Integer Least Squares approach.

## 5. Measurement Campaign and Trial Data Description

The methods previously described for jamming and spoofing detection and direction finding have been implemented and validated in laboratory conditions and then in open-field experiments. The open-field experimentation campaign was hosted at the *Technical Institute La Marañosa (ITM)* (Spain), a research and development organization belonging to the Spanish Department of Defense and managed by the National Institute of Aerospace Technology (INTA). A car equipped with the technological demonstrator of the concept described so far was driven along two outdoor areas belonging to the ITM Institute. These areas had visibility from the interference source:

- Location of the interference source (BaseTx). WGS-84 coordinates: $40^{\circ }16^{\prime }23.93^{\prime \prime }$ N, $3^{\circ }33^{\prime }55.30^{\prime \prime }$ W;
- Zone Z1. Straight trajectory within 1200 m from the source;
- Zone Z2. Curve trajectory within 100 m from the source.

The purpose of the technological demonstrator was to go one step further from the laboratory verification, completing the validation in real conditions (i.e., open-field with true GNSS signals and with radiated interference) and with real-time hardware acquisition (i.e., raw data have error sources inherent to acquisition: GNSS clock bias, imbalanced IQ channels, calibration needs, etc.).

### 5.1. Description of the Interference Sources

Interference sources specified for jamming and spoofing attacks were divided in two different types, the main features of which are detailed in Table 1. In both types, the jamming interference consisted of a single amplitude modulated continuous wave signal, generated and transmitted in real-time to the transmission device, implemented as an Universal Software Radio Peripheral (USRP) device. In the case of the spoofing interference source in configuration type 1, a first stage is carried out offline (i.e., no real-time transmission) and it consists of generation and pre-processing of the GNSS observables; subsequently, the pre-processed signal is transmitted through the USRP. In type 2, signal transmission was performed in real time through a multi-GNSS, multi-frequency signal generator.

### 5.2. Description of the Demonstrator

The technological demonstrator consists of hardware and software components installed on-board a ground vehicle moving in the areas affected by the interference. The demonstrator has three GNSS active antennas with an L-shaped layout. The preferred configuration for the estimation of DoA in the presence of spoofing are orthogonal baselines whose length is 1.5 m for the shortest baseline and 2 m for the longest one. Jamming configuration might be adapted without losing functionalities, but just reducing the performances of localization, thus antennas’ baselines in the demonstrator are defined according to the configuration that is optimal for the spoofing algorithms, in terms of orthogonality and distance between antennas.

This setup is equal for both jamming and spoofing tests, except for the hardware equipment used for the receiver stage. The receiver stage for spoofing detection is composed of two AsteRx4 GNSS MC/MF modules from Septentrio N.V. (each receiver supports up to two antennas). They share synchronization signals (10 MHz reference clock and Pulse Per Second (PPS) signal) between each other: this configuration is needed to form the synchronized SD and DD carrier-phase and pseudorange measurements.

The plan of the test cases execution is summarized in Table 2, which describes the main configuration of the completed trials. Note that these trials correspond to the autonomous D&AL mode defined in Section 2.

Combining straight line trajectories with different curve paths and static stages validates all the possible values of the DoA for both spoofing and jamming signals. The raw dataset recorded during trials is available in: https://zenodo.org/record/3532660#.XekN04jwanY. doi: 10.5281/zenodo.3532660.

## 6. On-Field Results

The results of processing of the datasets recorded during the live trials in the open field tests are reported in detail in this section. They prove the technological feasibility of a collaborative GNSS interference management concept for a civil aviation scenario, but also highlight the necessity of a deeply integrated design able to cope with the complexity of the system and of the possible scenarios.

### 6.1. Jamming Detection and Classification

#### 6.1.1. Jamming Detection Threshold Setting

**Figure 5:**
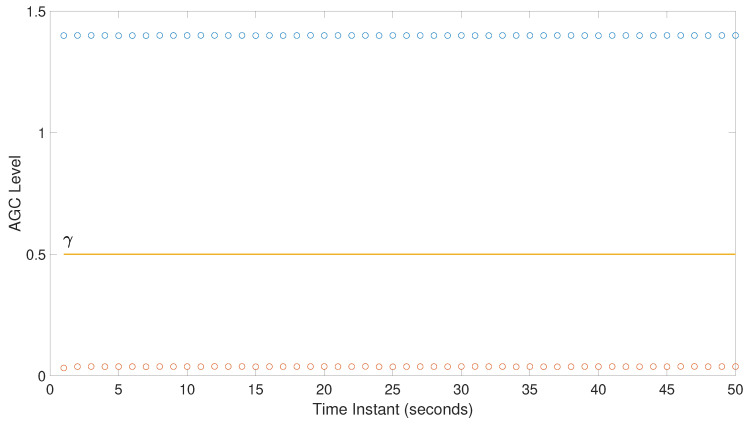
Example of test statistic comparison with AGC detector.

In the previous step of using the detectors, a threshold γ must be established for each method. γ will allow for discriminating among the different scenarios using the test statistic described in Section 3. Figure 5 shows the test statistic values obtained using the AGC test statistic method as an example for both interference free and OF-JAM.Z2D-Test 11 jamming scenario from Table 2. It shows that, as expected, the test statistic under jamming attack is much lower than the AGC test statistic in interference free scenario, since the received power is higher and the AGC has to decrease the gain in order to keep the power as stable as possible. γ is established after comparing statistically the test statistic values under both conditions for all the considered detectors.

#### 6.1.2. Jamming Detection and Direction Finding

**Figure 6:**
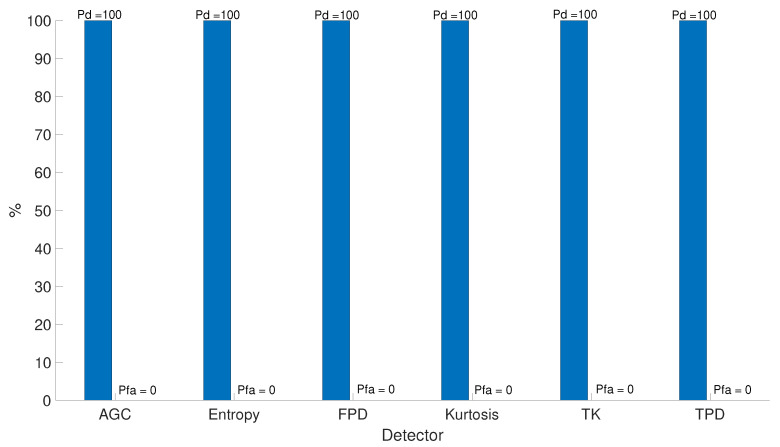
Detection results ($P_{d}$ + $P_{fa}$) for test scenario OF-JAM.Z2D-Test 11.

Results on jamming detection of on-field data are depicted in Figure 6. It shows the jamming detection results for test scenario OF-JAM.Z2D-Test 11, described in Section 5 using all the detectors described in Section 3. All the considered detectors with the current recorded tests show that they work perfectly well under realistic scenarios and they confirm the previous findings of the authors based on simulations and lab-tests as reported in [1,19,37]. In particular, the estimated Probability of Detection ($P_{d}$) is 100%, and the Probability of False Alarm ( $P_{fa}$) is 0. The reason for this high $P_{d}$ is because the jammer power set during the testing was set to a typical value for aircraft flying in the vicinity of the airport, namely around 50 dB-Hz, which is a very good range for a detector to operate with maximum performance. On its regard, this low ($P_{fa}$) is mainly due to two reasons: again because of the high power used during for the jammer, and also due to the relatively short recordings in time (up to about 400 s). Due to the recording time limitation, only a $P_{fa}$ of up to $10^{-5}$ can be measured (since the data processing is done ms by ms, and the maximum amount of data are about 400,000 ms for most of the scenarios in Table 2). It is most likely that the $P_{fa}$ is much lower, but it cannot be measured accurately with the provided amount of data. For this reason, the duration of the recordings were not enough for finding any false positive during the detection process.

For a trade-off $P_{fa}$-$P_{d}$ under simulated scenarios, the reader may refer to [21]; the work discussed here focuses on the actual measurement campaign with flying aircraft and the results are evaluated based on this realistic scenario.

#### 6.1.3. Jamming Classification

**Figure 7:**
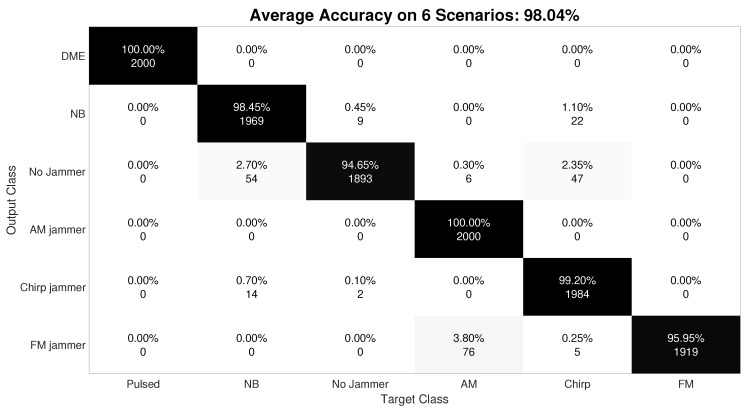
Confusion matrix with the classifier accuracy for in-lab data. The average accuracy is 98.04%.

In order to show a more complete performance analysis of the classification method, Figure 7 shows the confusion matrix for jamming classification with in-lab generated data. The additional in-lab generated data was added because the in-field measurements had only AM jammer, and thus a comprehensive classification with only one jammer type was not possible. We remark that the classification accuracy of AM jammer versus no jammer for field measurements was 100%. The confusion matrix in Figure 7 shows how accurate the classifier is in terms of how well it classifies and miss-classifies the test data set in the different classes it has.

Results show that the average accuracy of the detector is more than 98%. Pulsed and Amplitude Modulated (AM) classes are classified with no error after testing the classifier with 2000 images. Narrow Band (NB) class is miss-classified about 1.5% of cases. About 0.5% is classified as interference free, and 1.1% is classified as Chirp jammer (both jammer types spectrogram might look alike in the case of a low sweeping rate chirp signal). Regarding this, chirp jammer is also miss-classified 0.8% of cases, divided as 0.7% considered as NB and 0.1 as a No jammer scenario. Finally, the Frequency Modulated (FM) testing scenario is miss-classified for about 4% of cases. In addition, 3.8% is miss-classified as an AM jammer (both spectrogram look alike, especially in the case of single FM/AM tones), and 0.25% as a Chirp jammer.

### 6.2. Spoofing Detection and Direction Finding

The quality of the code and carrier phase measurements obtained from the GNSS receivers is critical in the determination of the performance reached by SpDDF. As detailed in [26,36], the presence of cycle slips in the carrier phase measurements is the symptom of a degraded quality of the affected measurements which must be discarded from the use in SpDDF: this can prevent the execution of the detection and direction finding algorithms. An algorithm able to detect presence of cycle slips and grant the quality of measurements is described in [26,36]. This avoids the use of bad measurements in SpDDF and has allowed for obtaining the results reported in this subsection. The tests hereafter are organized in ‘Static’ (OF-SPO-Z2-S-Tests 1 and 5 in Table 2) and ‘Dynamic’ (OF-SPO-Z2D-Tests 2, 4, and 6 in Table 2).

#### 6.2.1. Static Tests

**Figure 8:**
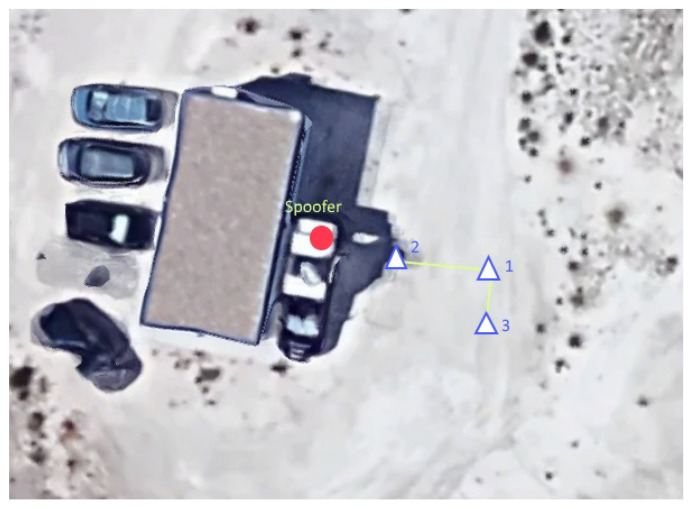
OF-SPO-Z2-S-Test 1 and 5: Open field static configuration of the transmitting and receiving antennas.

The layout of the three antennas used for the tests is described in Section 5. Figure 8 shows the location of the three antennas with respect to the spoofer during the static tests: the distance between the spoofer’s and vehicle’s antenna No. 2 is 3.5 m and the DoA of the counterfeit signal is known.

Table 3 shows an analysis of the behavior of the receivers and of the $D^{3}$ detector in the presence of the two spoofing attacks: all the counterfeit signals are tracked by the receivers and the whole subset is correctly detected. No cycle slips occur, and then the DDs are available for the spoofing detection during the entire test duration.

Since the true azimuth of the spoofer location with respect to the vehicle body frame is known in the static tests, a quantitative assessment of the DoA estimation algorithm performance can be performed. Thus, the main performance metrics of the whole SpDDF algorithm are summarized in Table 4, which reports the Root Mean Square (RMS), the Standard Deviation (STD), and some percentiles of the DoA estimation error (symbol $P_{k}$ indicates the k-th percentile). The DoA computed by the PAF algorithm is quite accurate for both the tests with only 1–2 degrees of error with respect to the correct value.

As a final remark about the static test, it must be noticed that the limited distance of the spoofer with respect to the receiving antennas makes this test configuration very demanding for the PAF algorithm because the DoA of the spoofing signal is not perfectly equal for all the three receiving antennas. Nevertheless, the algorithm was proved to be able to reach an acceptable level of accuracy even in these sub-optimal conditions.

#### 6.2.2. Dynamic Tests

**Figure 9:**
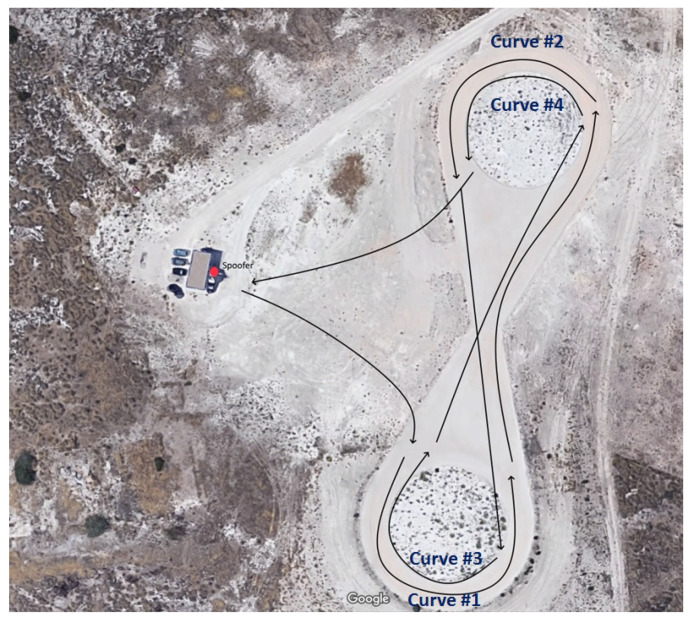
OF-SPO-Z2D Test 6: Spoofer location and vehicle trajectory.

According to Table 2, three datasets, namely OF-SPO-Z2D Test 2, OF-SPO-Z2D Test 4, and OF-SPO-Z2D Test 6 have been carried out in dynamic conditions. For the sake of brevity, the analysis is focused on OF-SPO-Z2D Test 6 only, where the trajectory driven by the vehicle equipped with the technological demonstrator is shown in Figure 9. The spoofer is set to transmit fake GPS signals on the L1 band and the path is covered at low speed (i.e., less than 50 km/h).

**Figure 10:**
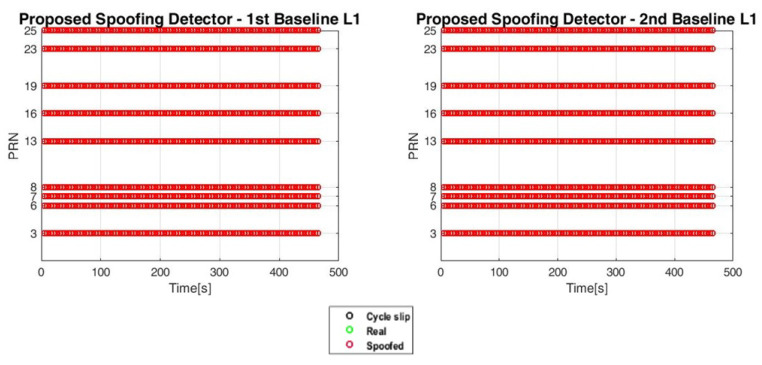
OF-SPO-Z2D Test 6: $D^{3}$ detection algorithm results. Detection is 100% correct.

**Figure 11:**
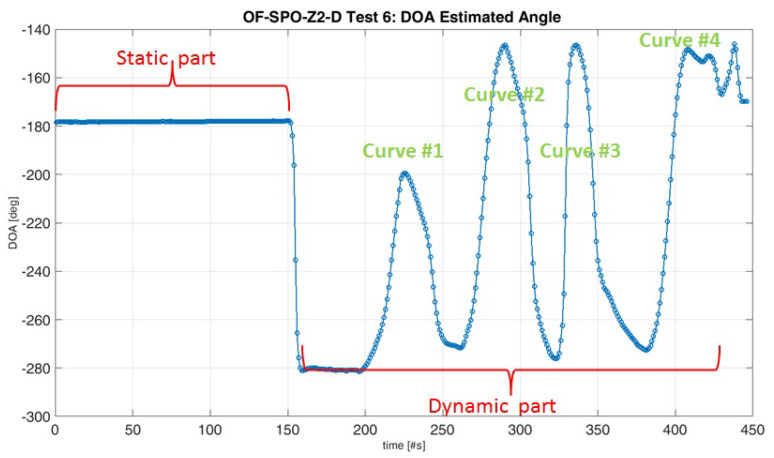
OF-SPO-Z2D Test 6: DoA estimation over the time.

The $D^{3}$ detection algorithm recognizes correctly all the spoofing signals for both the baselines, as it is evident looking at the results shown in Figure 10. As far as the DoA estimation results are concerned, Figure 11 proves that the test comprises an initial static phase where the estimated angle of arrival of the spoofed signal is constant, then the DoA varies continuously over the time, in appreciable accordance with the driven trajectory. This kind of qualitative analysis has been reported because a reliable reference system is not available due to the on-field nature of the tests and the presence of the spoofing attack, which hinders the use of a reference GNSS receiver.

## 7. Conclusions and Future Works

This paper presented a new interference management concept able to detect the presence of an intentional interference on the GNSS signals and locate its source. This interference management concept is mainly addressed to aviation applications, where the role of GNSS in the ATC is increasing and the safety risks related to jamming and spoofing attacks raise concerns and special attention. The methods to perform the detection and the localization have been presented for both jamming and spoofing attacks. The analysis of the results obtained during an on-field campaign allowed for assessing the performance of the proposed interference management concept and indicated the future developments that could pave the way for an effective adoption in ATC application.

Once the open-field experiments were executed and the results were analyzed and evaluated in accordance with the expected performances, some issues can be highlighted in order to justify the algorithms behavior, mainly: GNSS receiver clock bias estimation for spoofing direction finding and plane wave-front for jamming direction finding. These issues are not related with the algorithm itself, but to the hardware infrastructure used to record the raw input data used by the algorithms and the physical restrictions of the testing area. Taking into account all the limitations identified in the open field experiments and considering some issues that should be investigated still at algorithmic level and laboratory simulations, an evolution of the GNSS interference threats management concept and its benefits should be considered in further investigations.

Regarding the direction finding algorithms of jamming and spoofing, some potential benefits could be related to: the computation of the DoA measurement uncertainty, mitigation of error sources during signal acquisition, and reduction of the receiver clock bias. In addition, an optimized choice of the detection threshold and a more robust method to cluster the DD observables should be evaluated in order to improve the spoofing detection performance. Taking into account some of the improvements listed above, the algorithms could be upgraded and validated in new open-field experiments with GNSS interference radiation and longer jamming range to achieve plane wave-front. These experiments would consist of data acquisition and post-processing, as it has been done in the experiments presented in this paper. The definition of a prototype integrating the hardware for jamming and spoofing detection and direction finding, including the software for real-time processing simultaneous for jamming and spoofing, should also be considered for further experimentation campaigns. The improvements identified for the autonomous mode (D&AL) are also valid for the collaborative mode (D&CL). Therefore, it is worth planning future open-field trials to validate the D&CL mode. These trials would be identical to those described in Section 5 for D&AL mode, but using two demonstrators simultaneously moving with different paths. It will allow for comparing the localization capabilities of one single demonstrator (D&AL mode) with respect to multiple demonstrators (D&CL mode).

## Figures and Tables

**Table 1:** Interference sources characteristics of the open field tests.

Interference Source	Transmitter Configuration	Transmission Devices	Transmitted Signal	Antenna
Jamming	type 1	Laptop + USRP B205 mini + Amplifier Mini-Kits GALI-84M-R2-ENC	AM tone, $f_{c}=1577.00$ MHz	Straight fixed dipole Taoglas TLS.01.305111
	type 2	Laptop + USRP B205 mini + Amplifier GPS Networking LA20RPDC		Horn antenna A.H. Systems SAS-571
Spoofing	type 1	Laptop + USRP B205 mini + Amplifier Mini-Kits GALI-84M-R2-ENC	GPS L1 Spoofed location: Static at Sidney	Straight fixed dipole Taoglas TLS.01.305111
	type 2	Laptop + Signal generator Spirent GSS7700 + Amplifier GPS Networking LA20RPDC	GPS L1 Spoofed location: Static at Valencia	Horn antenna A.H. Systems SAS-571

**Table 2:** Open Field tests configuration. Legend: JAM: Jamming, SPO: Spoofing, Z1: Zone 1, Z2: Zone 2, D: Dynamic, S: Static.

Test Case ID	Duration	Transmitter Configuration	Receiver Mode	Vehicle Trajectory
OF-JAM-Z2D-Test 10	366 s	type 2	$f_{c}=1575.42$ MHz, $f_{s}=20$ Msample/s, 8 bits/sample, BW=20 MHz	Straight
OF-JAM-Z2D-Test 11	422 s			
OF-JAM-Z2D-Test 12	386 s			Curve
OF-JAM-Z2D-Test 13	139 s			
OF-JAM-Z2D-Test 14	67 s			
OF-SPO-Z2-S-Test 1	242 s	type 1	Configured to track only the spoofed signals	Static
OF-SPO-Z2-S-Test 5	260 s	type 2		
OF-SPO-Z2D-Test 2	301 s	type 1		Curve
OF-SPO-Z2D-Test 4	721 s	type 2		
OF-SPO-Z2D-Test 6	471 s			

**Table 3:** Static spoofing tests: spoofing detector performance.

Test Case ID	Spoofed PRNs Detected (%)
OF-SPO-Z2-S-Test 1	100
OF-SPO-Z2-S-Test 5	100

**Table 4:** Static spoofing tests: error statistics of DoA estimated by the direction finding algorithm.

Test Case ID	RMS (deg)	STD (deg)	$P_{50}$ (deg)	$P_{67}$ (deg)	$P_{90}$ (deg)	$P_{95}$ (deg)
OF-SPO-Z2-S-Test 1	2.8	0.08	2.81	2.85	2.9	3.02
OF-SPO-Z2-S-Test 5	1.2	0.05	1.22	1.45	1.8	1.9

## Appendix A. List of Acronyms

**ADS-B**	Automatic Dependent Surveillance-Broadcast
**AGC**	Automatic Gain Control
**AM**	Amplitude Modulated
**AoA**	Angle of Arrival
**ARAIM**	Advanced Receiver Autonomous Integrity Monitoring
**ASAS**	Airbone Separation Assurance System
**ATC**	Air Traffic Control
**ATM**	Air Traffic Management
$C/N_{0}$	Carrier-to-Noise Ratio
**CRPA**	Controlled Radiation Pattern Antenna
$D^{3}$	Dispersion of Double Differences
**DD**	Double Difference
**D&AL**	Detection and Autonomous Localization
**D&CL**	Detection and Collaborative Localization
**DFMC**	Dual-Frequency Multi-Constellation
**DoA**	Direction of Arrival
**DRSS**	Received Signal Strength Difference
**EGNOS**	European Geostationary Navigation Overlay Service
**ER**	Evolutionary Research
**EUROCAE**	European Organisation for Civil Aviation Equipment
**FDoA**	Frequency Difference of Arrival
**FM**	Frequency Modulated
**FPD**	Frequency Power Detector
**FRA**	Free Route Airspace
**GNSS**	Global Navigation Satellite System
**GPS**	Global Positioning System
**IATA**	International Air Transport Association
**ICAO**	International Civil Aviation Organization
**INTA**	National Institute of Aerospace Technology
**MLE**	Maximum Likelihood Estimation
**MOPS**	Minimum Operational Performance Standard
**NB**	Narrow Band
**PAF**	Precise and Fast
$P_{d}$	Probability of Detection
$P_{fa}$	Probability of False Alarm
**PLD**	Power Law Detector
**PPS**	Pulse Per Second
**PRN**	Pseudo Random Noise
**RFI**	Radio Frequency Interference
**RMS**	Root Mean Square
**SBAS**	Satellite-Based Augmentation System
**SD**	Single Difference
**SESAR**	Single European Sky Air traffic management Research
**SpDDF**	Spoofing Detection and Direction Finding
**STD**	Standard Deviation
**SV**	Space Vehicle
**SVM**	Support Vector Machine
**TDoA**	Time Difference of Arrival
**TF**	Time-Frequency
**TK**	Teager–Kaiser
**TPD**	Time Power Detector
**USRP**	Universal Software Radio Peripheral
**WAAS**	Wide-Area Augmentation System
